# COVID-19 vaccine uptake and associated factors among high school students in Lemi Kura Sub-City, Addis Ababa, 2022

**DOI:** 10.3389/fpubh.2025.1571217

**Published:** 2025-10-20

**Authors:** Etana Busa, Jemal Beksisa, Zinabu Reda, Sisay Abebe Debela

**Affiliations:** ^1^College of Health Sciences, Public Health School, Rift Valley University, Adama, Ethiopia; ^2^Ethiopian Public Health Institute, National Data Management Center, Addis Ababa, Ethiopia; ^3^Department of Public Health, College of Health Science, Salale University, Fiche, Ethiopia

**Keywords:** COVID-19 vaccine uptake, risk factors, Lemi Kura Sub-City, Addis Ababa, Ethiopia

## Abstract

**Background:**

Coronavirus disease 2019 (COVID-19) remains a major global health challenge, mostly affecting developing countries such as Ethiopia, which is facing low COVID-19 vaccination acceptance. This study aimed to assess the uptake of the COVID-19 vaccine and its associated factors among high school students in Lemi Kura Sub-City, Addis Ababa.

**Methods:**

An institution-based cross-sectional study was conducted at public high schools. Self-administered questionnaires were used to collect data from randomly selected high school students. We used Epi Info and SPSS for data entry and analysis. Descriptive and multivariable logistic regression analyses were performed to identify factors associated with COVID-19 vaccination.

**Results:**

Of the 802 participants (95% response rate), 46.2% were vaccinated. A total of 445 students (55.5%) had a positive attitude toward the COVID-19 vaccine, and 319 students (39.8%) had good knowledge about it. In addition, 625 students (78%) had undergone COVID-19 testing, and 286 students (35.7%) reported a history of infection. Factors significantly associated with vaccine uptake were good knowledge (AOR = 2.1, 95%CI: 1.4–3.1), positive attitude (AOR = 14.4, 95%CI: 9.5–22), prior testing (AOR = 2.1, 95%CI: 1.2–3.7), and absence of exposure to negative information (AOR = 2.26, 95%CI: 1.3–3.9).

**Conclusion:**

The overall COVID-19 vaccine uptake was low. Knowledge, attitude, prior COVID-19 testing, and exposure to misinformation significantly influenced vaccine uptake among high school students. Improving awareness among the target population is essential to enhancing vaccination rates.

## 1 Introduction

Coronavirus disease 2019 (COVID-19) remains a global health threat, leading to significant health, social, and economic impacts worldwide, including in Africa (1). As of 31 December 2021, the 47 countries in the World Health Organization (WHO) African Region had reported 7.1 million cases and 155,000 deaths, accounting for 2.5 and 2.9% of the global COVID-19 burden, respectively (2, 3). Africa was the last continent to be affected by the pandemic, and misconceptions about the virus continue to lead to low risk perception. In Ethiopia, over 495,998 cases and more than 5,000 deaths were reported as of October 2022 (4). Given the already strained health systems in the region, the actual burden of the disease may be more severe than what is reported.

Vaccination remains the most effective strategy for controlling COVID-19, offering life-saving protection worldwide (5, 6). In resource-limited settings such as Ethiopia, where shortages of trained healthcare personnel and medical equipment hinder treatment capacity, preventing viral transmission through vaccination is particularly critical. The WHO and its partners launched the COVID-19 Vaccines Global Access (COVAX) initiative in 2020 to accelerate vaccine development and manufacturing, ensuring fair and equitable access to vaccines and addressing global inequities (7). Initially, COVAX targeted 3% coverage, later expanding to 40% by the end of 2021 and 70% by mid-2022. While high-income countries (HIC) achieved these goals, low- and middle-income countries (LMIC), including Ethiopia, struggled due to limited supply, delivery delays, logistical challenges, and inequitable distribution (8, 9). By September 2021, only 2% of the population in low-income countries (LICs) had received at least one dose, compared to 65% in high-income countries (HICs) (10).

In Ethiopia, as of 27 March 2022, only 17.78% of the population had received the full dose of the COVID-19 vaccine, while 2.99% were partially vaccinated, resulting in the total coverage of 20.77% (11). National COVID-19 vaccination efforts have primarily targeted high-risk groups, including older adults and individuals in confined settings, such as prisons and schools, where disease transmission is expected to be high (12). Despite these efforts, studies across different population segments, including the general public, healthcare workers, university and college students, and older adults, have consistently reported low vaccine acceptance (13–18). Common barriers identified in these different studies include limited knowledge of vaccine benefits (14, 16, 19), negative information or misinformation mainly from social media (19, 20), and concerns about side effects (21, 22).

Young people represent a critical demographic for vaccination programs. According to the WHO classification, young people include adolescents (10–19 years old) and youth (15–24 years old) (23). In Ethiopia, this group, which primarily attends secondary school and college, constitutes nearly one-third of the population, making them a key target for COVID-19 vaccination. The majority of high school students fall within the 15–19 age range, aligning with the older adolescent category prioritized for vaccination, although older students may also be present due to the common occurrence of delayed school entry or slow academic progression in the country (24). High school students are particularly important because they are at a formative stage when lifelong health behaviors are established, they influence their peers and siblings, and they are highly engaged on digital platforms that can amplify both accurate information and misinformation.

Despite being a priority population, little is known about COVID-19 vaccine uptake and its associated factors among high school students in Ethiopia (11–13). Assessing vaccine uptake in this youth group is essential not only for achieving the national coverage target, which is still far below the 70% goal, but also for leveraging their influence to promote broader community acceptance. Therefore, this study aims to assess the magnitude of COVID-19 vaccine uptake and its associated factors among public high school students in Lemi Kura Sub-City, Addis Ababa, in 2022. The findings will inform strategies to improve vaccination coverage by strengthening access and enhancing behavioral change communication tailored to adolescents and youth.

## 2 Materials and methods

### 2.1 Study design and setting

We conducted an institution-based, cross-sectional survey in Lemi Kura Sub-City, Addis Ababa, from 1 June to 31 July 2022. Lemi Kura was officially established in October 2020 by incorporating land from the Bole and Yeka sub-cities. It is one of the fastest-growing areas of Addis Ababa, with a population of 366,922 in 2022, including 14,799 students enrolled in grades 9–12 across eight public and four private high schools (25). The sub-city encompasses both urban and formerly rural or peri-urban communities, resulting in a mix of socioeconomic and cultural backgrounds. This broader diversity provides an important context for studying COVID-19 vaccine uptake among young people.

Geographically, Lemi Kura Sub-City shares approximately 75–580% of its border with the Oromia region (formerly rural and peri-urban areas, now part of the newly established Shager City), specifically to the east, north, and south, and is bordered to the west by Bole and Yeka sub-cities. The area is characterized by rapid urbanization and widespread informal settlements. Historically rural areas, such as Woreda 2 and Woreda 14, where agriculture was once the main source of income, have undergone significant transformation with expanding urbanization (26). This peri-urban transition has influenced educational access and grade progression, contributing to the presence of older students in the area’s high schools. This situation is further shaped by contextual factors such as late school enrollment, grade repetition, and disruptions to schooling during the COVID-19 pandemic, all of which have delayed academic progression. In addition, socioeconomic challenges common in peri-urban communities, including limited educational access and the need for children to contribute to household livelihoods, also affect the timing of school attendance.

### 2.2 Study population

The source population included all high school students attending public schools in Lemi Kura Sub-City. The study specifically targeted regular students in grades 9–12 who were aged 18 years and above during the 2022 academic year, as this group was eligible to receive any of the available COVID-19 vaccine types. As noted in the Study Design and Setting, older students (18 years and above) are commonly enrolled in these grades due to factors such as late school enrollment, grade repetition, and disrupted educational progression. Students younger than 18 years old and those with serious mental or physical illnesses at the time of data collection were excluded.

### 2.3 Sample size and sampling procedure

The sample size was determined using the single population proportion formula, assuming a 5% margin of error, a 95% confidence level (*α* = 0.05), and a 50% prevalence of COVID-19 vaccine uptake. After accounting for a 10% non-response rate and applying a design effect of two, the final sample size was 844.

**Figure 1:**
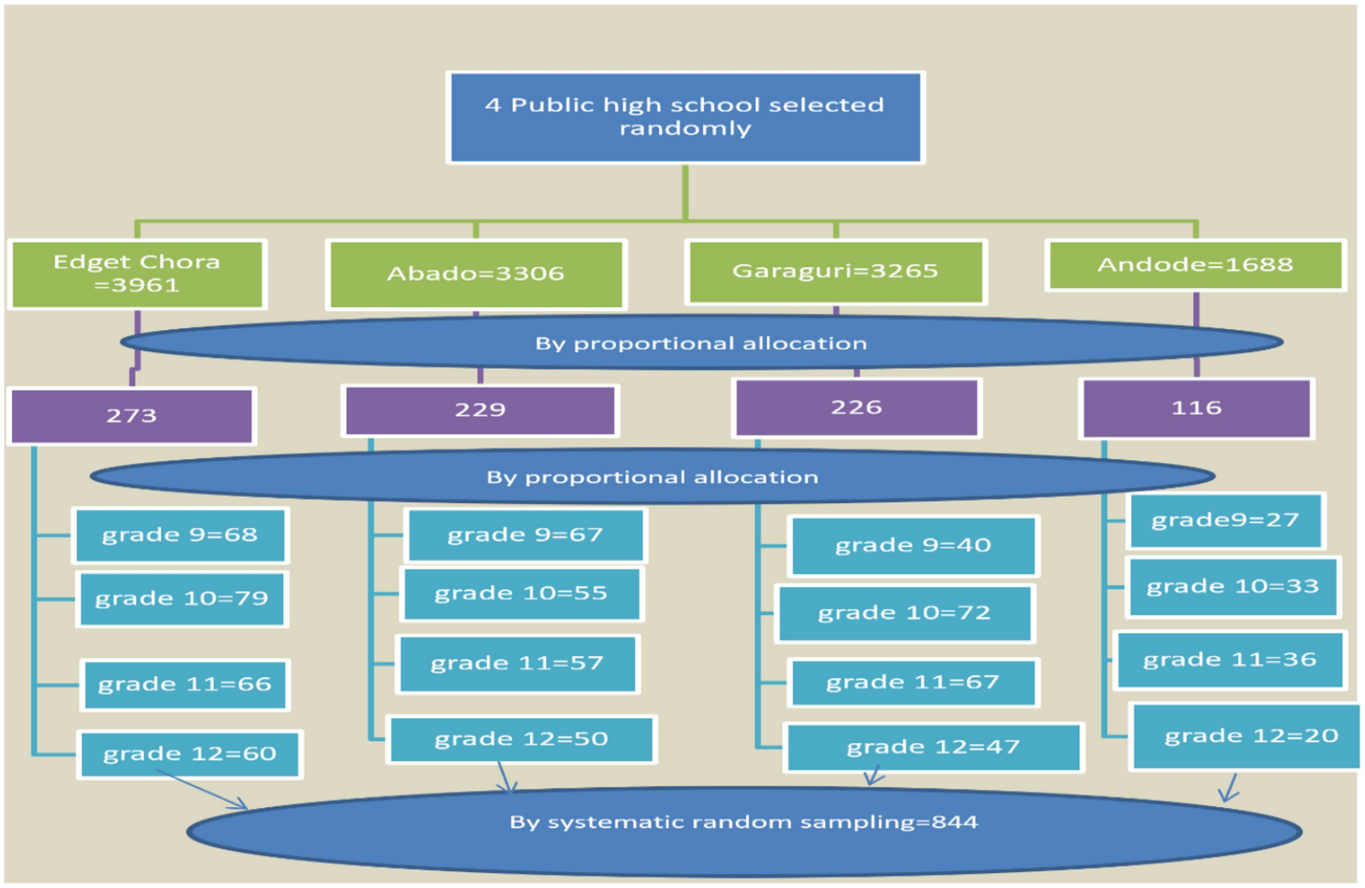
Schematic diagram of the sampling procedure for the study on COVID-19 vaccine uptake among high school students in Lemi Kura Sub-City, Addis Ababa, 2022.

A multistage sampling technique was employed to select study participants. First, four public high schools—Edget Chora, Abado, Beshale, and Andode—were randomly selected. The number of participants was proportionally allocated to each selected school and grade level, and then students were randomly selected for the study (Figure 1).

### 2.4 Study variables

The dependent variable was COVID-19 vaccination status. The independent variables included sociodemographic characteristics, knowledge, attitudes, perceptions, beliefs, and exposure to COVID-19- and vaccine-related misinformation.

### 2.5 Data collection procedure and quality assurance

Data were collected using a structured, self-administered questionnaire adapted from relevant studies and the WHO tools designed to assess knowledge, attitudes, and perceptions toward COVID-19 vaccines, which include the Community Needs, Perceptions, and Demand Assessment Tool, which evaluates barriers, community vulnerabilities, and attitudes toward vaccinations, and the COVID-19 vaccination survey, a standardized instrument for identifying drivers of vaccine decisions, including knowledge, attitudes, and practices (27, 28). The questionnaire was translated into Amharic and back-translated into English to ensure consistency.

Four trained nurses, recruited through the local health bureau in collaboration with school administrations based on their prior experience in community- and school-based health programs, facilitated the data collection under the supervision of two senior nurses. They had no personal or professional relationship with the researchers outside of the study, and their role was limited to following the study protocol and minimizing potential bias. To maintain data quality, training was provided for data collectors and supervisors, the data collection tool was pretested prior to use, and daily checks for completeness and consistency were conducted by the principal investigator and supervisors.

### 2.6 Operational definition

- Vaccine Uptake: The act of eligible individuals receiving the COVID-19 vaccine.
- Knowledge: Awareness and understanding of key aspects of the COVID-19 vaccine, including its development, effectiveness, safety, and potential side effects.
- Good knowledge: Score above the mean on the knowledge assessment.
- Poor knowledge: Score at or below the mean.
- Attitudes: Perceptions, beliefs, and willingness of individuals regarding the vaccine’s safety, necessity, fairness, and promotion.
- Positive attitude: Score above the mean on attitude-related questions.
- Negative attitude: Score at or below the mean.
- Perception: The individual’s perception of COVID-19 risk, both personally and for others, along with the likelihood of future infections.
- Belief: Personal beliefs regarding immunity to COVID-19 and vaccine effectiveness.
- Exposure: Personal or family exposure to misinformation about COVID-19 testing, infection, and vaccine.

### 2.7 Data processing and analysis

The data were coded, cleaned, and entered into Epi Info version 3.5.1, and then exported to SPSS version 20 for analysis. Descriptive statistics, including frequencies and proportions, were calculated. Bivariate and multivariable logistic regression analyses were conducted to identify factors associated with vaccine uptake. Variables with a *p*-value of ≤ 0.2 in the bivariate analysis were included in the multivariable model after checking multicollinearity and model fitness (29, 30). Multicollinearity among the independent variables was evaluated using the variance inflation factor (VIF), and all the variables included in the final model had VIF values below the commonly accepted threshold of 10. *p*-values less than 0.05 in the multivariable analysis model were considered statistically significant.

## 3 Results

### 3.1 Sociodemographic characteristics of the respondents

A total of 802 students participated in this study, yielding a 95% response rate. Among the respondents, 54.7% (439) were male individuals, and 56.6% (454) were aged 18–19 years old. Approximately 90.3% (724) resided in urban areas, and 58.7% (471) identified as Orthodox Christians (Table 1).

**Table 1:** Sociodemographic characteristics of high school students in public schools in Lemi Kura Sub-City, Addis Ababa, Ethiopia, 2022.

Variables	Categories	Frequency	Percentage
Age (Year)	18–19 years old only	439	54.7
	> = 20 years	363	45.3
Residence	Urban	724	90.3
	Rural	78	9.7
Sex	Male individuals	454	56.6
	Female individuals	348	43.4
Religion	Orthodox	471	58.7
	Protestant	185	23.1
	Muslims	100	12.5
	Other	46	5.7
Education	Secondary /high school	802	100

### 3.2 Knowledge and attitude of the respondents regarding the COVID-19 vaccine

Of the five questions assessing knowledge of the COVID-19 vaccine, 678 respondents (84.5%) recognized the risks of receiving an excessive amount of vaccine, while 552 (68.8%) were aware that the vaccine was developed to combat the pandemic. Overall, 319 respondents (39.8%) demonstrated good knowledge, scoring above the mean (Table 2).

**Table 2:** Knowledge of high school students about the COVID-19 vaccine, Lemi Kura Sub-City, Addis Ababa, Ethiopia, 2022.

Variable	Category	Frequency	Proportion
Do you know about the development of the COVID-19 vaccine?	Yes	552	68.8
	No	250	31.2
Do you know about the effectiveness of the COVID-19 vaccine?	Yes	448	55.9
	No	354	44.1
Is it dangerous to receive an excessive amount of the COVID-19 vaccine?	Yes	678	84.5
	No	124	15.5
Does the COVID-19 vaccine increase allergic reactions?	Yes	397	49.5
	No	405	50.5
Does the vaccine increase autoimmune diseases?	Yes	370	46.1
	No	432	53.9
Vaccine knowledge status	Good	319	39.8
	Poor	483	60.2

Of the six questions assessing attitudes toward the COVID-19 vaccine, 321 respondents (40%) believed in its safety, while 387 (48.3%) recognized its importance. In addition, 533 respondents (66.4%) supported fair vaccine distribution. Overall, 445 respondents (55.5%) had a positive attitude, scoring above the mean, while 357 (44.5%) had a negative attitude (Table 3).

**Table 3:** Attitudes toward the COVID-19 vaccine among high school students, Lemi Kura Sub-City, Addis Ababa, Ethiopia, 2022.

Attitudes	Strongly Agree or agree *N* (%)	Neutral *N* (%)	Strongly disagree or disagree *N* (%)
Is the newly discovered COVID-19 vaccine safe?	321 (40)	119 (14.8)	362 (45.1)
Is the COVID-19 vaccine essential for us?	387 (48.3)	65 (8.1)	350 (43.6)
COVID-19 vaccines developed in Europe and America are safer than those made in other countries	321 (40)	110 (13.7)	371 (46.3)
Would you encourage your family/friends/relatives to get vaccinated?	368 (45.9)	63 (7.9)	368 (45.9)
It is not possible to reduce the incidence of COVID-19 without vaccination	367 (45.7)	56 (7.0)	379 (47.2)
The COVID-19 vaccine should be distributed fairly to all of us	533 (66.4)	67 (8.4)	202 (25.2)
Attitude score level	Positive attitude	445 (55.5)	
	Negative attitude	357 (44.5)	

### 3.3 Perceptions, beliefs, and exposure regarding COVID-19 and its vaccination

The respondents displayed diverse perceptions, beliefs, and exposure regarding COVID-19 and its vaccination. While nearly half (48%) of the participants believed that they were not at risk of contracting COVID-19 in the future, a significant proportion (35.7%) reported no concern about the virus. However, 35.8% recognized COVID-19 as a major risk to Ethiopia, although only 19% perceived it as a major personal risk. Immunity perceptions varied among respondents, with 43.4% believing they had some level of immunity. Opinions regarding vaccination were divided; only 27.7% agreed that the vaccine was effective, while 28.4% disagreed. COVID-19 exposure was notable, with 35.7% of the respondents having tested positive and 50.4% reporting a family member’s positive test. Negative information about COVID-19 vaccination was widely circulated, with 85.2% of the respondents having encountered such information (Table 4).

**Table 4:** Perceptions, beliefs, and exposure regarding COVID-19 and its vaccination among high school students in Lemi Kura Sub-City, Addis Ababa, Ethiopia, 2022.

Variables	Categories *N* (%)					Total
How likely do you think it is that you will get COVID-19 in the future?	Extremely	Very likely	Moderate	Slightly	Not at all	802 (100)
	77 (9.6)	14 (1.7)	162 (20.2)	164 (20.4)	385 (48)	
Overall, how worried are you about COVID-19?	Extremely	Very	Not very	Some what	Not at all	802 (100)
	148 (18.5)	23 (2.9)	263 (32.8)	80 (10)	286 (35.7)	
To what extent do you think COVID-19 poses a risk to people in Ethiopia?	Major risk	Moderate	Minor	Slightly	No risk at all	802 (100)
	287 (35.8)	226 (28.2)	150 (18.7)	103 (12.8)	36 (4.5)	
I think I have some level of immunity to COVID-19	Strongly agree	Agree	Neutral	Disagree	Strongly disagree	802 (100)
	138 (17.2)	348 (43.4)	158 (19.7)	131 (16.3)	27 (3.4)	
COVID-19 vaccines may be effective in protecting me from COVID-19	Strongly agree	Agree	Neutral	Disagree	Strongly disagree	802 (100)
	63 (7.9)	222 (27.7)	156 (19.5)	228 (28.4)	132 (16.5)	
To what extent do you think COVID-19 poses a risk to you personally?	Major risk	Moderate	Minor	Slightly	No risk at all	802 (100)
	152 (19)	237 (29.6)	266 (33.2)	0(0)	147 (18.3)	
Do you know if you have had or currently have COVID-19?	Definitely had it	Definitely not had it	Probably had it	Probably not had it	–	802 (100)
	74 (9.2)	399 (49.8)	154 (19.2)	175 (21.8)		
Have you been tested for COVID-19?	Positive	Negative	Not tested	–	–	802 (100)
	286 (35.7)	339 (42.3)	177 (22.1)			
Have your family members been tested for COVID-19?	Positive	Negative	Not tested	–	–	802 (100)
	404 (50.4)	291 (36.3)	107 (13.3)			
Have your friends been tested for COVID-19?	Positive	Negative	Not tested	–	–	802 (100)
	179 (22.3)	459 (57.2)	164 (20.4)			
Have you ever heard negative information about the COVID-19 vaccination?	Yes	No	–	–	–	802 (100)
	683 (85.2)	119 (14.8)				

### 3.4 Magnitude of COVID-19 vaccine uptake

**Figure 2:**
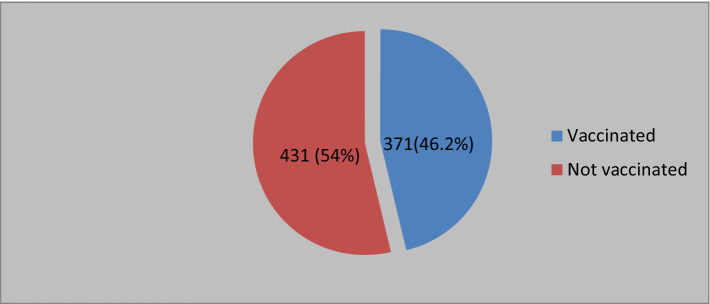
COVID-19 vaccination among high school students of Lemi Kura Sub-City, Addis Ababa, Ethiopia, 2022.

Regarding COVID-19 vaccine uptake, 371 respondents (46.2%) had received the COVID-19 vaccine, while 431 (53.7%) had not voluntarily agreed to be vaccinated (see Figure 2).

### 3.5 Factors associated with Addis Ababa, Ethiopia COVID-19 vaccine uptake

To identify the factors associated with COVID-19 vaccine uptake, the variables that showed an association in the bivariate analysis with a *p*-value ≤ 0.2 were included in the multivariable analysis. The bivariate analysis identified several factors as potential predictors, including age, knowledge about the COVID-19 vaccine, attitude toward the vaccine, perceived risk of infection, concern about COVID-19, prior testing for COVID-19, having a friend or family member tested for COVID-19, and exposure to negative information about the vaccine. In the multivariable analysis, knowledge about the COVID-19 vaccine (AOR = 2.1, 95%CI: 1.4–3.1), attitude toward the COVID-19 vaccine (AOR = 14.4, 95%CI: 9.5–22), prior COVID-19 testing (AOR = 2.1. 95%CI: 1.2–3.7), and exposure to negative information about the COVID-19 vaccine (AOR = 2.26 95%CI: 1.3–3.9) showed a statistically significant association with vaccine uptake (*p* < 0.05) (Table 5).

**Table 5:** Factors associated with COVID-19 vaccine uptake among high school students in public schools in Lemi Kura Sub-City, Addis Ababa, Ethiopia, 2022 (*N* = 802).

Variables	Category	COVID-19 vaccine uptake		COR (95% CI)	AOR (95% CI)
		Yes	No		
Age in years	18–19 years old	178 (48)	185 (42.9)	0.82 (0.61, 1.1)	0.84 (0.58, 1.2)
	> = 20 years	193 (52)	246 (57.1)	1	1
Vaccine knowledge status	Good knowledge	208 (56.1)	111 (25.8)	3.67 (2.73, 4.1)	2.10 (1.43, 3.08)*
	Poor knowledge	163 (43.9)	320 (74.2)	1	1
Attitude score level	Positive attitude	322 (87.0)	120 (28.0)	17.3 (11.9, 24.1)	14.42 (9.55, 22.08)*
	Negative attitude	48 (13.0)	309 (72.0)	1	1
How likely do you think it is that you will get COVID-19 in the future?	Extremely likely	34 (9.2)	43 (10.0)	0.51 (0.14, 1.8)	1.36 (0.21, 8.65)
	Moderate	91 (24.5)	71 (16.5)	0.31 (0.09, 1.03)	1.14 (0.19, 6.85)
	Not at all	152 (41.0)	233 (54.1)	0.61 (0.18, 1.1)	1.30 (0.22, 7.49)
	Slightly	90 (24.3)	74 (17.2)	0.33 (0.1, 1.1)	0.96 (0.16, 5.68)
	Very likely	4 (1.1)	10 (2.3)	1	1
Overall, how worried are you about COVID-19?	Extremely	84 (22.6)	64 (14.9)	0.41 (0.16, 1.01)	1.54 (0.43, 5.55)
	Not at all	154 (41.5)	132 (30.8)	0.45 (0.19, 1.11)	1.23 (0.35, 4.25)
	Not very	94 (25.3)	169 (39.4)	0.96 (0.39, 2.34)	2.32 (0.66, 8.10)
	Somewhat	31 (8.4)	49 (11.4)	0.84 (0.32, 2.22)	2.13 (0.56, 8.16)
	Very	8 (2.2)	15 (3.5)	1	1
Do you think that COVID-19 poses a risk to people in Ethiopia?	Major risk	154 (41.5)	133 (30.9)	0.48 (0.23, 1.0)	1.17 (0.42, 3.29)
	Minor risk	79 (21.3)	71 (16.5)	0.51 (0.24, 1.07)	0.79 (0.27, 2.30)
	Moderate risk	90 (24.3)	136 (31.6)	0.85 (0.41, 1.77)	1.24 (0.44, 3.49)
	Slightly	35 (9.4)	68 (15.8)	1.09 (0.49, 2.42)	2.46 (0.81, 7.44)
	No risk at all	13 (3.5)	23 (5.3)	1	1
Do you know if you have had or currently have COVID-19?	Definitely had it	44 (11.9)	30 (7.0)	0.62 (0.36, 1.06)	0.84 (0.39, 1.80)
	Definitely not had it	173 (46.6)	226 (52.4)	1.17 (0.82, 1.68)	1.37 (0.85, 2.22)
	Probably had it	71 (19.1)	83 (19.3)	1.05 (0.68, 1.62)	1.21 (0.66, 2.20)
	Probably not had it	83 (22.4)	92 (21.3)	1	1
Have you been tested for COVID-19?	Positive	172 (46.4)	114 (26.5)	3.3 (2.3, 5.44)	2.1 (1.17, 3.75)*
	Negative	144 (38.8)	195 (45.2)	1.61 (1.1, 2.4)	1.54 (0.88, 2.73)
	Not tested	55 (14.8)	122 (28.3)	1	1
Have your family members been tested for COVID-19?	Positive	217 (58.5)	187 (43.4)	0.46 (0.29, 0.71)	0.87 (0.44, 1.71)
	Negative	117 (31.5)	174 (40.4)	0.8 (0.5, 1.2)	1.11 (0.55, 2.24)
	Not tested	37 (10.0)	70 (16.2)	1	1
Have your friends been tested for COVID-19?	Positive	90 (24.3)	89 (20.6)	0.76 (0.5, 1.2)	1.02 (0.55, 1.88)
	Negative	210 (56.6)	249 (57.8)	0.9 (0.62, 1.3)	1.17 (0.69, 1.98)
	Not tested	71 (19.1)	93 (21.6)	1	1
Have you ever heard negative information about the COVID-19 vaccination?	Yes	308 (83.0)	375 (87.0)	1	1
	No	63 (17.0)	56 (13.0)	1.4 (0.92, 2.02)	2.26 (1.31, 3.89)*

* Significantly associated at a *p*-value of <0.05.

## 4 Discussion

High school youth represent a crucial group for public health research and interventions. This developmental stage is a formative period for establishing lifelong health behaviors that can affect long-term outcomes, such as the risk of chronic diseases and reproductive health (31). Importantly, high school students have an influential role that extends beyond their personal choices; within households and peer networks, they often act as role models, shaping the behaviors and attitudes of younger individuals (32). Therefore, assessing vaccination behavior in high school students is essential, as interventions targeting this group can generate immediate health benefits while fostering sustainable, community-wide impacts.

This study revealed that COVID-19 vaccine uptake was 46.2%, meaning that more than half of the population either refused or hesitated to receive the vaccine. This finding is consistent with the studies conducted in Wolaita Sodo, Ethiopia (45.5%) (15), the pooled prevalence in Ethiopia (51.6%) (13), Egyptian medical students (49%) (22), and nursing students across seven countries (43.8%) (33). However, our finding is lower than those reported in the studies conducted in Dessie, Ethiopia (56%) (18), Sudan (55.1%) (34), and West India (79%) (35). It is, however, higher than the findings reported in Gonder, Ethiopia (34.1%) (17), Jimma, Ethiopia (27.2%) (16), Uganda (37.3%) (36), and Jordan (29.4%) (20).

The observed discrepancies in vaccine uptake across studies may be explained by differences in sociodemographic and socioeconomic factors. While the majority of comparative studies examined university or college students, our study focused on high school students (16, 18, 34, 36). University students generally have better access to reliable health information and services than high school students do. Similarly, youth from urban and higher-income families are more likely to afford transportation to vaccination sites and benefit from greater exposure to awareness campaigns, whereas those from rural or low-income households often face barriers such as limited health facilities and lower trust in government programs (10, 35, 37). In addition, cultural and religious beliefs, which vary across regions, further shape parental and peer attitudes toward vaccination. In our study, only 46.2% of the participants were willing to be vaccinated, which is significantly lower than the 70% global vaccination goal set by the WHO and COVAX to control the pandemic (7). The novelty of COVID-19, coupled with misinformation and conspiracy theories, likely contributed to this low uptake, with 87% of the unvaccinated respondents citing negative information from social media and peers as their primary reasons. In this study, less than half of the eligible students were vaccinated, leaving a significant proportion at risk of infection. The low COVID-19 vaccine uptake presents a major challenge to controlling the pandemic. Vaccine uptake has been a global concern for decades, and the situation has become even more complex with COVID-19, largely due to misinformation and conspiracy theories (15, 16, 22, 38).

Students with good knowledge about the COVID-19 vaccine were 2.1 times more likely to get vaccinated, while those with a positive attitude toward the vaccine were 14.4 times more likely to receive the vaccine compared to their counterparts. This indicates that increasing knowledge and awareness can significantly improve vaccine uptake. Similar findings were reported in studies conducted at Wolkite and Jimma Universities in Ethiopia (16, 19). Furthermore, the students with a positive attitude toward the vaccine were less likely to be influenced by misinformation, and this finding is supported by the studies conducted in the Gonder community, among healthcare workers in the Democratic Republic of Congo, and a systematic review in Ethiopia (17, 19, 38).

In addition, the students who had previously tested positive for COVID-19 infection were 2.1 times more likely to receive the vaccine than those who had never been tested. This suggests that experiencing the illness firsthand heightened their awareness of its severity, motivating them to take preventive measures such as vaccination. Similar findings were reported in the studies conducted in Wolaita Sodo, Southern Ethiopia, and the Gonder community, Northwestern Ethiopia (15, 17). While this positive association is important, it should also be noted that some individuals may hold the opposing belief that natural infection provides sufficient protection, leading them to avoid vaccination. Considering both perspectives provides a more comprehensive understanding of how prior infection shapes vaccine acceptance.

Moreover, the students who were not exposed to misinformation about COVID-19 vaccines were 2.26 times more likely to be vaccinated than those who had encountered misinformation. Alarmingly, 85% of the high school students in this study reported being exposed to negative information about COVID-19 vaccines, highlighting the urgent need for targeted public health interventions. Previous studies have shown that misinformation is a key driver of vaccine hesitancy among young people and can significantly undermine vaccine confidence (39, 40). To address this issue, targeted interventions are recommended, including school-based awareness campaigns, peer-led education, and the integration of vaccination into student health services. Additionally, leveraging student clubs and using social media platforms can be considered as effective approaches to counter misinformation and promote vaccine acceptance among youth (40, 41).

### 4.1 Limitations of the study

This study has some limitations. The use of self-administered questionnaires may have introduced response bias, and the cross-sectional design limited the ability to establish causal relationships between the variables. To mitigate these issues, the questionnaire was pilot-tested on 5% of the sample to ensure clarity and consistency, and the study participants were clearly informed about the purpose of the study to minimize misunderstandings and enhance the reliability of their responses.

## 5 Conclusion and recommendations

This study revealed that COVID-19 vaccine uptake among high school students was low, despite the disease being a major public health problem. Factors such as knowledge and attitude toward the vaccine, prior COVID-19 testing, and exposure to negative information about the vaccine were identified as significant risk factors.

Therefore, it is crucial to enhance awareness among high school students through Behavior Change Communication (BCC), health education, and outreach vaccination programs. Collaboration with various stakeholders is essential to promote COVID-19 vaccination and increase its adoption.

## Data Availability

The raw data supporting the conclusions of this article will be made available by the authors, without undue reservation.
